# Functional comparison of antisense proteins of HTLV-1 and HTLV-2 in viral pathogenesis

**DOI:** 10.3389/fmicb.2013.00226

**Published:** 2013-08-07

**Authors:** Benoit Barbeau, Jean-Marie Peloponese, Jean-Michel Mesnard

**Affiliations:** ^1^Département des sciences biologiques and Centre de recherche BioMed, Université du Québec à MontréalMontréal, QC, Canada; ^2^Centre d’études d’agents Pathogènes et Biotechnologies pour la Santé, Université Montpellier 1Montpellier, France; ^3^UMR5236, Centre d’études d’agents Pathogènes et Biotechnologies pour la Santé, Centre National de la Recherche ScientifiqueMontpellier, France

**Keywords:** HTLV-1, HTLV-2, antisense transcription, HBZ, APH-2

## Abstract

The production of antisense transcripts from the 3′ long terminal repeat (LTR) in human T-lymphotropic retroviruses has now been clearly demonstrated. After the identification of the antisense strand-encoded human T-lymphotropic virus type 1 (HTLV-1) bZIP (HBZ) factor, we reported that HBZ could interact with CRE-binding protein (CREB) transcription factors and consequently turn off the important activating potential of the viral Tax protein on HTLV-1 5′ LTR promoter activity. We have recently accumulated new results demonstrating that antisense transcripts also exist in HTLV-2, -3, and -4. Furthermore, our data have confirmed the existence of encoded proteins from these antisense transcripts (termed antisense proteins of HTLVs or APHs). APHs are also involved in the down-regulation of Tax-dependent viral transcription. In this review, we will focus on the different molecular mechanisms used by HBZ and APH-2 to control viral expression. While HBZ interacts with CREB through its basic zipper domain, APH-2 binds to this cellular factor through a five amino acid motif localized in its carboxyl terminus. Moreover, unlike APH-2, HBZ possesses an N-terminal activation domain that also contributes to the inhibition of the viral transcription by interacting with the KIX domain of p300/CBP. On the other hand, HBZ was found to induce T cell proliferation while APH-2 was unable to promote such proliferation. Interestingly, HTLV-2 has not been causally linked to human T cell leukemia, while HTLV-1 is responsible for the development of the adult T cell leukemia/lymphoma. We will further discuss the possible role played by antisense proteins in the establishment of pathologies induced by viral infection.

## INTRODUCTION

The human T-lymphotropic virus type 1 (HTLV-1) was the first pathogenic retrovirus to be isolated in humans (Poiesz et al., 1980; Miyoshi et al., 1981). This virus is the etiological agent of adult T cell leukemia/lymphoma (ATLL) and HTLV-1-associated myelopathy/tropical spastic paraparesis (HAM/TSP). Although an important number of individuals are infected by HTLV-1, only 10% will eventually develop pathologies (Barbeau and Mesnard, 2007; Matsuoka and Jeang, 2007). Leukemic cells in ATLL are mostly CD4^+^ T cells. One of the best studied associations between HTLV-1 and oncogenesis is the viral Tax protein, as demonstrated in primary T cells and various mouse models (Matsuoka and Jeang, 2007). It likely results from the capacity of Tax to activate multiple transcription factors and to induce genetic instability (Mesnard and Devaux, 1999; Boxus et al., 2008). However, Tax is often repressed in cells from ATLL patients (Takeda et al., 2004; Satou et al., 2006). Selective pressure mediated by strong anti-Tax immune response might justify the lack of Tax expression in ATLL cells (Hanon et al., 2000; Enose-Akahata et al., 2012). HTLV-2 is closely related to HTLV-1 and shares most viral genes such as Tax and Rex but HTLV-2 is clinically distinct from HTLV-1 since it is not associated with any forms of leukemia (Feuer and Green, 2005). However, HTLV-2 can efficiently immortalize and transform T lymphocytes *in vitro* (Ye et al., 2003) and HTLV-2 Tax can immortalize human CD4^+^ T cells at higher efficiency than HTLV-1 Tax (Imai et al., 2012). Taken together, these data suggest that Tax expression is not sufficient for ATLL development and is thus dispensable at least for the late stage of leukemogenesis. Additional viral proteins obviously are thereby needed to play such a role. Indeed, growing evidence indicate that antisense transcripts produced from the 3′ long terminal repeat (LTR) of the HTLV-1 proviral DNA might fit such a role. These transcripts are involved in the production of the HTLV-1 bZIP (HBZ) factor in infected cells (Cavanagh et al., 2006; Murata et al., 2006). We have recently demonstrated that such antisense transcription also exists in HTLV-2-infected cells and permits the synthesis of the antisense protein of HTLV-2 (APH-2; Halin et al., 2009). This review will discuss the role of antisense transcription and resulting viral proteins in the development of pathologies associated with HTLV infection.

## ANTISENSE TRANSCRIPTS ARE PRODUCED FROM THE 3′ LTR OF THE HTLV PROVIRAL DNA

**FIGURE 1:**
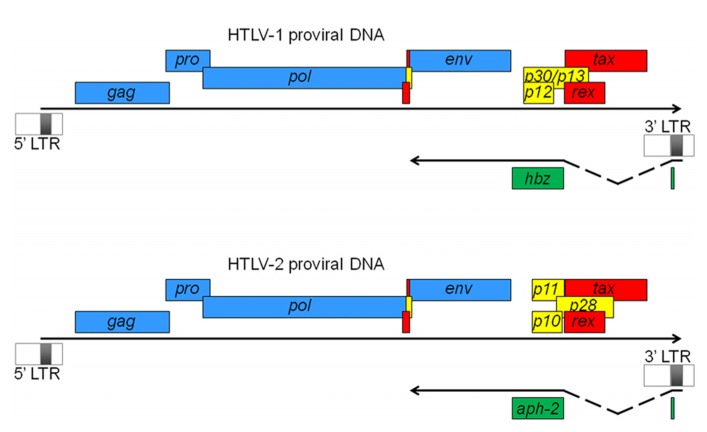
**HTLV-1 and HTLV-2 proviral DNA.** Viral ORFs are depicted as either common retroviral proteins (blue), regulatory factors (red), or accessory proteins (yellow).****Antisense strand-encoded ORFs are also indicated (green). Antisense transcripts are initiated from the 3′ LTR and spliced to produce the antisense proteins, HBZ and APH-2. In both cases, transcripts are initiated at a similar position in the 3′ LTR (which constitutes the first exon) and are spliced to the second exon, which contains the main ORF region as well as the 3′ untranslated region. Their polyA tail is positioned 1450 nucleotides from the ORF stop codon. In HTLV-1, an unspliced transcript initiating downstream of the 3′ LTR (not shown here) has also been described (Yoshida et al., 2008).

HTLV-1 and HTLV-2 are complex retroviruses sharing a similar genome structure with an approximate 70% nucleotide sequence homology (Feuer and Green, 2005). Like all retroviruses, they harbor essential genes for their replication, which include *gag*, *pol* and *env*. In addition to these genes, they harbor genes coding for the Tax and Rex regulatory proteins (**Figure 1**). Tax acts in trans to activate transcription initiating from the viral promoter in the 5′ LTR and Rex regulates viral gene expression post-transcriptionally by facilitating cytoplasmic shuttling of incompletely spliced viral mRNAs. HTLV-1 encodes the accessory proteins p12, p13, and p30 whereas HTLV-2 encodes the p10, p11, and p28 accessory gene products (**Figure 1**). Studies have indicated that these proteins are dispensable for *in vitro* infection and transformation of T cells but are important for the ability of the virus to infect, spread, and persist *in vivo* (Albrecht and Lairmore, 2002; Feuer and Green, 2005). Transcription of all these viral genes is dependent on the 5′ LTR region, which is segmented in three regions termed U3, R, and U5. The U3 region harbors important elements like the Tax-responsive elements (TxREs). A Tax dimer interacts with cellular Activating Transcription Factor/CRE-binding (ATF/CREB) proteins bound specifically to TxREs (Basbous et al., 2003b; Nyborg et al., 2010). The formation of such a complex on the 5′ LTR then serves as a binding site for the recruitment of the pleiotropic cellular coactivators p300/CBP through their interaction with Tax. Recruitment of p300/CBP to the viral promoter induces local nucleosome modification by histone acetylation and facilitates stable binding of components of the basal transcription machinery allowing the stimulation of viral transcription (Gachon et al., 2002; Luebben et al., 2010).

For a long time, all retroviral genes have been thought to be transcribed through the U3 region in the 5′ LTR of the provirus. However, the production of antisense transcripts from the 3′ LTR in HTLV-1-infected cell lines (Larocca et al., 1989) and the presence of a conserved open reading frame (ORF) in the complementary strand of the HTLV-1 provirus (Larocca et al., 1989; Gaudray et al., 2002) suggested the existence of viral mRNA of negative polarity. In 2002, we clearly demonstrated the existence of an antisense strand-encoded protein termed HBZ in HTLV-1-infected cell lines (Gaudray et al., 2002). Further experiments by our teams and others have revealed that the antisense HBZ-encoding transcript was spliced and polyadenylated (Cavanagh et al., 2006; Murata et al., 2006; Satou et al., 2006; Yoshida et al., 2008) and that different HBZ isoforms could be produced, with one of them being the most abundant and dependent on a spliced transcript (**Figure 1**). In 2009, we also characterized a spliced antisense mRNA in HTLV-2-infected cell lines, involved in the production of APH-2 (Halin et al., 2009). This transcript is structured similarly to the HBZ transcript, i.e., it is spliced, initiates in the 3′ LTR at multiple sites, and is polyadenylated. The length of the intron and of the 5′ and 3′ untranslated regions is also similar to that of the HBZ transcript (**Figure 1**). These similarities could suggest that the expression of antisense transcription in the HTLV (and STLV) retrovirus family has been conserved among the different viruses. Indeed, we have recently confirmed the synthesis of antisense proteins from HTLV-3 and -4 proviral DNA (Larocque et al., 2011). On the other hand, unlike HBZ mRNA, we did not observe alternative splicing for APH-2, -3, and -4 mRNAs (Halin et al., 2009; Larocque et al., 2011) suggesting that these retroviral antisense genes are likely producing a single isoform.

## HBZ AND APH-2 INHIBIT Tax-DEPENDENT VIRAL TRANSCRIPTION

**FIGURE 2:**
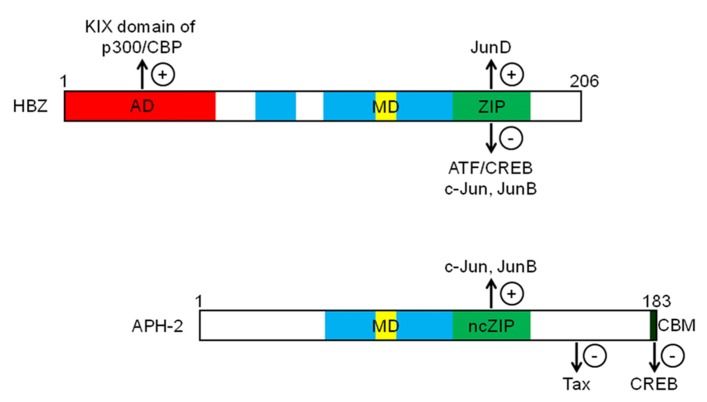
**Schematic representations of HBZ and APH-2 functional domains.** Both proteins contain basic domains (blue) involved in their nuclear localization. HBZ possesses an N-terminal activation domain (AD in red), a modulatory domain (MD in yellow) and a ZIP (green) controlling the transcriptional activity of Jun and ATF/CREB proteins. While APH-2 does not contain any activation domain, it can interact with c-Jun and JunB through a non-canonical ZIP region (ncZIP in green) and with CREB through a C-terminal CREB-binding motif (CBM in black). Unlike HBZ, APH-2 binds to Tax (+) and (-) respectively indicate an activating or inhibiting effect of viral proteins on targeted proteins.

Initial studies highlighted the negative impact of HBZ expression on HTLV-1 replication by virtue of its capacity to inhibit Tax-mediated activation of HTLV-1 transcription (Gaudray et al., 2002; Arnold et al., 2006). HBZ acts as a repressor of viral transcription by forming heterodimers with CREB, CREB-2, CREM, and ATF-1 that are no longer capable of binding to TxRE. HBZ is a nuclear transcriptional factor able to interact with ATF/CREB proteins (Gaudray et al., 2002; Lemasson et al., 2007; Hagiya et al., 2011) through its basic zipper (bZIP) domain (**Figure 2**), leading to inhibition of their DNA-binding activity (Hivin et al., 2005, 2006). Consequently, Tax cannot be positioned on the viral promoter and is thereby unable to trans-activate HTLV-1 transcription (Gaudray et al., 2002; Lemasson et al., 2007). Interestingly, APH-2 has also been shown to repress Tax-mediated viral transcription (Halin et al., 2009; Yin et al., 2012) by interacting with CREB. However, unlike HBZ, this interaction is mediated by the LXXLL motif present in the C-terminus of APH-2 (**Figure 2**; Yin et al., 2012). Interestingly, it has been shown that the repressive activity of APH-2 is less strong than that of HBZ. This difference in the inhibitory potential of both proteins might be explained by the additional interaction of HBZ with p300/CBP. Unlike APH-2, HBZ possesses a transcriptional activation domain within its N-terminal region (**Figure 2**) involved in an interaction with the KIX domain of p300/CBP (Clerc et al., 2008; Cook et al., 2011). In the context of viral transcription, we have demonstrated that HBZ could displace p300/CBP from the HTLV-1 promoter by competing with Tax for binding to the KIX domain (Clerc et al., 2008). Moreover, this mechanism appears more efficient than that of the ZIP domain in mediating repression of Tax-dependent viral transcription. On the other hand, additional reports have demonstrated that HBZ can activate cellular gene transcription through its interaction with p300/CBP (Kuhlmann et al., 2007; Polakowski et al., 2010; Macaire et al., 2012).

Analyses of transcription factors involved in the regulation of HTLV-1 antisense transcription showed that the Sp1 transcription factor was critical for its trans-activation (Yoshida et al., 2008; Gazon et al., 2012). We recently demonstrated that Sp1 enhanced antisense transcription by cooperating with the HBZ–JunD heterodimer (Gazon et al., 2012), which implicates the previously reported ZIP-dependent interaction between HBZ and JunD (Thébault et al., 2004; **Figure 2**). Thus, HBZ not only inhibits expression of other viral proteins such as Tax but also stimulates its own expression. While the majority of HTLV-1-specific CD8^+^ T cells recognize the Tax protein, the frequency of HBZ-specific CD8^+^ T cells is significantly lower (Macnamara et al., 2010). HBZ-expressing cells could thus escape lysis from a cytotoxic T lymphocyte (CTL) response and would consequently enhance viral persistence in infected people (Suemori et al., 2009; Macnamara et al., 2010). It has also been suggested that Tax can stimulate HBZ expression although this issue remains controversial, as most results were obtained from cell lines transfected with a Tax expression plasmid and reporter vectors containing only one HTLV-1 LTR (Larocca et al., 1989; Cavanagh et al., 2006; Yoshida et al., 2008; Landry et al., 2009). Indeed, such an effect by Tax has not been confirmed in T cells infected with a complete proviral DNA containing two LTRs (Belrose et al., 2011). Moreover, we have observed that activation of sense transcription from the 5′ LTR down-regulated the synthesis of antisense transcripts from the 3′ LTR (Cavanagh et al., 2006; Landry et al., 2007). It should also be emphasized that the regulation of APH-2 expression in infected cells remains currently unknown.

## HBZ AND APH-2 REGULATE THE AP-1 PATHWAY IN A DIFFERENT MANNER

In unstimulated T cells, basal protein levels of the AP-1 complex are low but there is a rapid induction of AP-1 activity after T cell stimulation. The AP-1 transcription complex has been shown to be involved in the regulation of numerous cellular genes involved in lymphocyte activation. The AP-1 transcription factor consists of heterodimers between the Jun (c-Jun, JunB, and JunD) and Fos (c-Fos, FosB, Fra-1, and Fra-2) family members through their ZIP domains. AP-1 complexes are not limited to Jun and Fos dimers, since Jun and Fos proteins have been shown to dimerize with other bZIP proteins, including members of the ATF/CREB family and the Maf transcription factors. ATLL cells have been shown to contain constitutively activated AP-1 complexes, which are mainly constituted of JunD (Mori et al., 2000). HBZ, via its ZIP domain, forms heterodimers with all the members of the Jun family (Basbous et al., 2003a; Thébault et al., 2004). The interaction of HBZ with c-Jun and JunB results in repression of their transcriptional activity through degradation or sequestration into transcriptionally inactive nuclear bodies (Basbous et al., 2003a; Matsumoto et al., 2005; Hivin et al., 2007; Clerc et al., 2009). On the other hand, the HBZ/JunD heterodimer can activate transcription of cellular genes such as the human telomerase catalytic subunit (hTERT) and the antiapoptotic Bfl-1 (Kuhlmann et al., 2007; Macaire et al., 2012). HBZ can also dysregulate other cell-signaling pathways such as FoxP3 (Satou et al., 2011), NF-κB (Zhao et al., 2009; Zhi et al., 2011; Wurm et al., 2012), TGF-β (Zhao et al., 2011), and Wnt pathways (Ma et al., 2012). The resulting dysregulated pathways suggest that HBZ expression might play a central role in the development of ATLL and HAM/TSP through these altered transcription factors (Mesnard et al., 2006; Satou et al., 2006, 2011; Arnold et al., 2008; Zhi et al., 2011; Sugata et al., 2012).

Unlike HBZ, analysis of APH-2 does not predict a typical bZIP domain. However, APH-2 does interact with c-Jun and JunB through its non-canonical bZIP domain (**Figure 2**; Marban et al., 2012). Although inconsistently, APH-2 does not appear to bind to JunD *in vivo* (Marban et al., 2012). However, given the unique structure of its ZIP domain it is not surprising that APH-2 does not form canonical interactions with human bZIPs (Reinke et al., 2010). An interesting outcome from the interaction between APH-2 and the different Jun family members is that APH-2 potentiates their trans-activation activity (Marban et al., 2012). This is thereby in sharp contrast to the negative or positive modulation of HBZ on the various Jun factors. Interesting results from this study have also inferred a potential complex between Tax2B and APH-2 involving a region from amino acid 102 to 183. As no competition occurred between Jun factors and Tax2B in their binding to APH-2, the region of interaction for Tax2B is likely located outside the bZIP-like domain (**Figure 2**). Furthermore, the modulation of AP-1 activation by Tax2B was greatly reduced when APH-2 was co-expressed. However, the above experiments were conducted in overexpression condition and it will therefore be mandatory to confirm these data with physiologically relevant expression levels. These results highlight a very complex interplay between Tax 2 and APH-2 in relation to other transcription factors and it will be important to determine how this type of interaction is affected in the context of ATF/CREB family members. Furthermore, we have not observed that HBZ interacts with Tax (Hivin et al., 2005) and therefore further experiments will be required to address this issue.

## HBZ INDUCES IL-2-INDEPENDENT T CELL PROLIFERATION

Based on the effect of HBZ on AP-1-dependent gene expression, HBZ might contribute to the dysregulation of cell proliferation in infected cells. In fact, two reports using HBZ-specific shRNA expression vectors have demonstrated that HBZ was important for the proliferation of HTLV-1-infected cell lines (Satou et al., 2006; Arnold et al., 2008). In these studies, although HBZ was not found to be required for HTLV-1-induced peripheral blood mononuclear cell (PBMC) immortalization, HBZ was shown to be important for cell proliferation of infected cells and had an impact in infection experiments in rabbits. Furthermore, along with these studies, other reports have determined that HBZ expression correlated with proviral DNA load, likely through its capacity to permit infected cells to proliferate (Arnold et al., 2008; Li et al., 2009). Based on these reports, HBZ has further been associated with ATLL development. Early implication of HBZ in ATLL development has been based on the occurrence of HBZ expression in ATLL cells from most tested patients (Satou et al., 2006; Usui et al., 2008). This is in contrast to Tax, whose expression is often repressed in ATLL cells. A balance between Tax and HBZ expression (Barbeau and Mesnard, 2011) might thereby be important to permit cells to proliferate and, in fact might be determinant in hampering previously reported Tax-mediated cell senescence (Zhi et al., 2011). More recent evidence has also permitted to suggest mechanisms by which HBZ could be implicated in ATLL, such as its induction of hTERT expression via a Sp1-dependent mechanism (Kuhlmann et al., 2007), its interaction with ATF-3 to suppress the ATF-3-induced p53 transcription activity stimulation (Hagiya et al., 2011), and the inhibition of p300/CBP acetyl transferase activity (Wurm et al., 2012). Additional data also argue for a potential oncogenic property of HBZ. Indeed, experiments in transgenic mice expressing HBZ in CD4^+^ T cells resulted in high incidence of T cell lymphoma (Satou et al., 2011). In addition, NOD/SCID mice engrafted with HBZ-silenced HTLV-1-infected cells were less infiltrating and formed tumors at lesser extent than control infected cells (Arnold et al., 2008). We have also recently demonstrated that proviral DNA-expressed HBZ induced anchorage-independent growth in NIH 3T3 cells and correlated with induced JunD expression (Gazon et al., 2012). An intriguing observation also suggests that the HBZ transcript itself could be essential for induced IL-2-independent T cell proliferation (Satou et al., 2006). Kinetics and intracellular compartmentalization study of HTLV-1 mRNA expression is indirectly favoring the implication of such a transcript in cell proliferation, as HBZ viral transcripts have been shown strongly sequestered in the nucleus when compared to other viral transcripts (Rende et al., 2011). However, these data have been obtained after transfection of HLtat cells with the HTLV-1 proviral clone ACH. When 293T cells were similarly transfected, such a nuclear retention of HBZ mRNA was not confirmed (Li et al., 2012) showing that this observation strongly depends on the transfected cell line and has to be confirmed in physiologically relevant conditions.

Recent studies have compared the proliferation-inducing capacity of APH-2 to this functional characteristic of HBZ (Douceron et al., 2012). Although not associated with any forms of leukemia, HTLV-2 has nonetheless been linked to lymphocytosis in infected patients (Bartman et al., 2008). Certain similar features, reminiscent of a potential impact on cell proliferation, were noted when similar analyses were performed on APH-2. First, APH-2 expression has been detected in most PBMC samples of HTLV-2-infected patients (Halin et al., 2009; Douceron et al., 2012). Second, a correlation between APH-2 expression levels and proviral DNA load was also noted (Douceron et al., 2012). Furthermore, a typical nuclear sequestration of its mRNA was inferred in both patient cells and infected cell lines (Bender et al., 2012). However, unlike HBZ, APH-2 is not capable of leading to IL-2-independent growth of IL-2-dependent cell lines (Douceron et al., 2012). Moreover, the absence of an impact on proliferation was further suggested in a study demonstrating no impact on proliferation of infected cells lines in addition to no effect on the immortalization capacity of HTLV-2 on infected PBMCs (Yin et al., 2012). In conclusion, no clear correlation between APH-2 mRNA expression, its nuclear retention, and HTLV-2-induced lymphocytosis was observed.

## PERSISTENCE OF HTLV-1 VS. HTLV-2 INFECTION IN RELATION TO THEIR RESPECTIVE ANTISENSE PROTEIN

Using the rabbit model, a number of elegant studies have focussed on the consequence of HBZ silencing on HTLV-1 replication. Although no drastic differences were noted in HTLV-1 infection and replication in cell culture conditions, infection of rabbits with irradiated HTLV-1-producing cells highlighted an important role of HBZ on HTLV-1 persistence, as indicated by anti-HTLV-1 antibody titers and proviral DNA loads (Arnold et al., 2006). When similar experiments were conducted with HTLV-2, again APH-2 did not alter viral replication in cell culture experiments. However, inoculation of rabbit with irradiated cells producing HTLV-2 virions deficient for APH-2 led to a higher and more sustained antibody response correlating with higher proviral load at certain time points (Yin et al., 2012). These results thereby argue that, unlike HBZ, APH-2 is not necessary for viral persistence but might instead be related to the reduced capacity of HTLV-2 infection.

**FIGURE 3:**
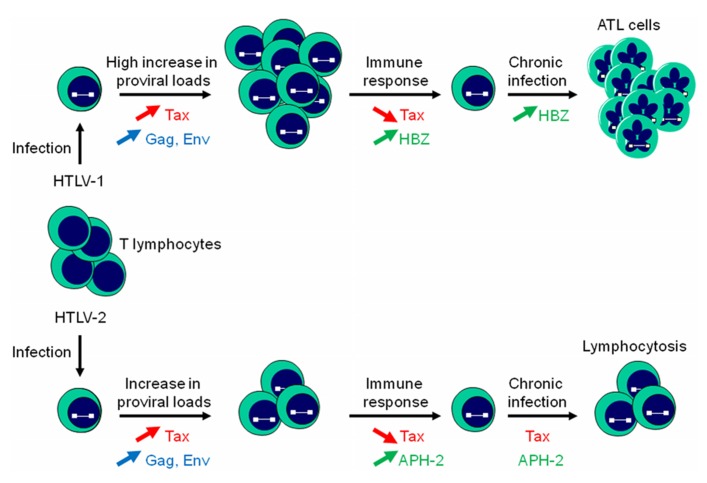
**HBZ and APH-2 differently impact T cell proliferation.** Infection of T lymphocytes with HTLV-1 or HTLV-2 results in an increase in proviral loads, with a more pronounced effect for HTLV-1 proviral DNA load. An antibody response against Gag and Env and Tax-specific CTL responses induces killing of infected cells. HTLV-1 can evade the immune response by reducing Tax and stimulating HBZ expression. HBZ would subsequently promote the establishment of a chronic infection by inhibiting Tax-dependent viral transcription, stimulating its own expression, and inducing T cell proliferation. APH-2 also down-regulates Tax-dependent HTLV-2 transcription but is unable to induce cell proliferation. A low expression of Tax induced by APH-2 through its capacity to positively modulate c-Jun activation could be responsible for HTLV-2-stimulated lymphocytosis.

Both HTLV-1 and HTLV-2 are able to establish a persistent infection in CD4^+^ and CD8^+^ T cells but HTLV-1 establishes a more robust infection in T lymphocytes (Kannian et al., 2012). Moreover, infection results in an increase in proviral loads and viral expression such as Tax, Gag, and Env (**Figure 3**). However, cells highly expressing viral proteins are eliminated by the humoral response and CTL activity of the host (Macnamara et al., 2010; Enose-Akahata et al., 2012; Enose-Akahata et al., 2013). At this stage, HBZ and APH-2 might play a crucial role by down-regulating Tax-dependent viral transcription and may allow infected cells to evade the immune response. In additionally, HBZ promotes the proliferation of infected T lymphocytes (**Figure 3**). This dual action probably confers a survival advantage on HBZ-expressing cells and is consistent with the observation that HBZ favors the establishment of persistent infection in HTLV-1-inoculated rabbits (Arnold et al., 2006). On the other hand, unlike HBZ, APH-2 is not required for viral persistence (Yin et al., 2012) and unable to promote lymphocytosis (Douceron et al., 2012). However, a low expression of Tax from the TxREs of the 5′ LTR induced by AP-1 transcriptional activity stimulated by APH-2 could explain lymphocytosis commonly observed in HTLV-2 carriers (**Figure 3**). Further experiments are definitely needed to better understand the mechanism by which HTLV-2 infection could lead to lymphocytosis.

## CONCLUSIONS AND PERSPECTIVES

The discovery of HBZ has led to important new research avenues with potential association with HTLV-1-associated diseases. In fact, ATLL cells from infected patients consistently express HBZ and substantial evidence points toward its implication in viral persistence and ATLL cell survival. Recent evidences further suggest that HBZ lead toward a Treg phenotype. Future experiments will be needed to determine if similar attributes can be given to APH-2. As HBZ and APH-2 also differ mechanistically and functionally in their interaction with transcription factors such as Jun and CREB, it will be important to further expand on these differences and define the exact nature of these interactions. Several transcription factors known to interact with HBZ will also need to be analyzed in the context of APH-2 and in relation to the different cellular localizations of these proteins. In addition, the role played by HBZ on proliferation needs to be clarified as to how its transcript impacts IL-2-independent growth. These studies might also consider variation in the sequence of APH-2 transcripts, which could explain their different ability in inducing proliferation. Finally, these experiments should also be undertaken with equivalent antisense proteins of HTLV-3 and HTLV-4, i.e., APH-3 and APH-4 to determine how these proteins alter the various functions associated to HBZ. Finally, these comparative studies will provide a better understanding as to the mechanism by which HBZ is involved in ATLL development, which might also lead to new potential ATLL treatment.

## Conflict of Interest Statement

The authors declare that the research was conducted in the absence of any commercial or financial relationships that could be construed as a potential conflict of interest.
